# Feasibility of deploying peer coaches to mentor frontline home health aides and promote mobility among individuals recovering from a stroke: pilot test of a randomized controlled trial

**DOI:** 10.1186/s40814-022-00979-4

**Published:** 2022-01-31

**Authors:** Penny H. Feldman, Margaret V. McDonald, Nicole Onorato, Joel Stein, Olajide Williams

**Affiliations:** 1 Center for Home Care Policy & Research, Visiting Nurse Service of New York, 220 East 42nd Street, New York, NY 10017 USA; 2 Department of Rehabilitation and Regenerative Medicine, Columbia University Vagelos College of Physicians and Surgeons, Department of Rehabilitation Medicine, Weill Cornell Medicine, New York-Presbyterian Hospital, 180 Ft. Washington Ave., Harkness Pavilion Room 1-165, New York, NY 10032 USA; 3 Department of Neurology, Columbia University, 710 West 168th Street, New York, NY 10032 USA

**Keywords:** Intervention feasibility, Intervention fidelity, Stroke rehabilitation, Post-stroke mobility, Home health aides, Peer coaches, Pilot randomized controlled trial

## Abstract

**Background:**

Each year, approximately 100,000 individuals receive home health services after a stroke. Evidence has shown the benefits of home-based stroke rehabilitation, but little is known about resource-efficient ways to enhance its effectiveness, nor has anyone explored the value of leveraging low-cost home health aides (HHAs) to reinforce repetitive task training, a key component of home-based rehabilitation. We developed and piloted a Stroke Homehealth Aide Recovery Program (SHARP) that deployed specially trained HHAs as “peer coaches” to mentor frontline aides and help individuals recovering from stroke increase their mobility through greater adherence to repetitive exercise regimens. We assessed the feasibility of SHARP and its readiness for a full-scale randomized controlled trial (RCT). Specifically, we examined (1) the practicability of recruitment and randomization procedures, (2) program acceptability, (3) intervention fidelity, and (4) the performance of outcome measures.

**Methods:**

This was a feasibility study including a pilot RCT. Target enrollment was 60 individuals receiving post-stroke home health services, who were randomized to SHARP + usual home care or usual care only. The protocol specified a 30-day intervention with four planned in-home coach visits, including one joint coach/physical therapist visit. The primary participant outcome was 60-day change in mobility, using the performance-based Timed Up and Go and 4-Meter Walk Gait Speed tests. Interviews with participants, coaches, physical therapists, and frontline aides provided acceptability data. Enrollment figures, visit tracking reports, and audio recordings provided intervention fidelity data. Mixed methods included thematic analysis of qualitative data and quantitative analysis of structured data to examine the intervention feasibility and performance of outcome measures.

**Results:**

Achieving the 60-participant enrollment target required modifying participant eligibility criteria to accommodate a decline in the receipt of HHA services among individuals receiving home care after a stroke. This modification entailed intervention redesign. Acceptability was high among coaches and participants but lower among therapists and frontline aides. Intervention fidelity was mixed: 87% of intervention participants received all four planned coach visits; however, no joint coach/therapist visits occurred. Sixty-day follow-up retention was 78%. However, baseline and follow-up performance-based primary outcome mobility assessments could be completed for only 55% of participants.

**Conclusions:**

The trial was not feasible in its current form. Before progressing to a definitive trial, significant program redesign would be required to address issues affecting enrollment, coach/HHA/therapist coordination, and implementation of performance-based outcome measures.

**Trial registration:**

ClinicalTrials.gov, NCT04840407. Retrospectively registered on 9 April 2021

## Key messages regarding feasibility

- What uncertainties existed regarding the feasibility?
  - Uncertainty regarding the following:
    - ▪ Practicability of participant recruitment, enrollment, and randomization procedures
    - ▪ Intervention feasibility and acceptability to program participants
    - ▪ Fidelity to key intervention components—e.g., Would planned coach visits be completed? Would joint visits be scheduled to facilitate coordination among coaches, physical therapists, and frontline aides?
    - ▪ The feasibility of validated outcome measures to assess the primary participant outcome—improved post-stroke mobility
- What are the key feasibility findings?
  - The intervention as originally designed was not feasible. To achieve target study enrollment, investigators had to relax the eligibility criterion that required all eligible participants have a frontline HHA to be coached. This in turn required intervention redesign such that coaches worked directly with participants rather than as peer mentors to frontline aides.
  - Acceptability and fidelity to the redesigned intervention were high among coaches and participants but less so among frontline aides and participants’ physical therapists. Joint visits among coaches, therapists, and frontline aides did not occur as planned, limiting coordinated efforts to promote participant exercise and mobility.
  - Only 55% of participants completed the baseline and follow-up performance-based mobility measures, physical and environmental constraints imposed by participants’ personal circumstances, and living conditions interfered with successful completion by the remaining 45%.
- What are the implications of the feasibility findings for the design of the main study?
  - Absent significant program redesign, the intervention is not ready for a full-scale effectiveness trial. Issues affecting participant enrollment, coordination among coaches and other members of the care team, and implementation of performance-based outcome measures limited the feasibility of both the intervention and research protocol.

## Background

Stroke is a leading cause of disability in the United States of America (USA) and the single most important cause of severe disability among community-dwelling adults [1]. Each year, approximately 795,000 new and recurring strokes occur, with estimated direct and indirect costs of nearly $50 billion [1]. Individuals recovering from stroke are the second most common users of publicly funded post-acute rehabilitation services across all settings. They account for more than 475,000 people a year [2]. Approximately 100,000 individuals receive home-based post-stroke services paid for by Medicare, the health insurance program for US residents 65 years of age or older [3]. Mobility impairment from stroke—difficulty in rising, transferring, walking, and related risk of falls—remains one of the costliest and most disabling deficits of the disease, disproportionately affecting older persons and racial-ethnic minorities [4–11]. For many individuals, improved mobility is therefore a primary goal of rehabilitation [12].

Evidence from trials, systematic reviews, and meta-analysis has shown that intensive, repetitive task training, such as sit-to-stand and walking practice, leads to improvement in walking distance and speed, sit-to-stand, and activities of daily living that require moving from one place to another [12–17]. Prescribed by a therapist, similar exercises repeated at home have been shown to have the same impact as facility-based training on mobility at 12 months [18]. Rehabilitation at home gives individuals the opportunity to practice skills and develop compensatory strategies in their own surroundings, leading to improved capacity to perform daily activities and increased social participation and quality of life [15, 19–23]. Compared to more costly institutional rehabilitation, it also provides greater encouragement and support for a family-centered model of stroke rehabilitation [20, 23].

A meta-analysis of 20 randomized controlled trials found that just 16 h of augmented exercise therapy in the first 6 months post-stroke yielded significant gains in activities of daily living and lower limb function [19]. However, many individuals in the home care setting infrequently perform their therapist-prescribed exercises outside of their regularly scheduled physical therapy sessions [24, 25]. Because exercise regimens ideally are designed to accommodate an individual’s physical and cognitive limitations, poor adherence may be due to insufficient motivation or caregiver support [26–28]. Systematic reviews attest to the dearth of studies on resource-efficient ways to enhance adherence to exercise regimens in the steadily growing post-acute home health care sector [29–31].

Medicare-funded home health services include physical therapy and skilled nursing care to promote post-stroke recovery. Additionally, as needed, many individuals are assigned a home health aide (HHA) who spends several hours a day in their home to provide personal assistance in transferring, walking, and performing other personal and household tasks. These frontline aides may be an untapped resource with the potential to enhance the effectiveness of home-based rehabilitation therapy. To tap into this resource, however, would require that selected aides receive additional training and support beyond the Medicare-required 75 h of entry-level training, with its limited focus on therapeutic activities or stroke [32]. This would likely be impractical given high training costs, the need for intensive hands-on skill building, and the challenge of finding substitute aides to provide ongoing assistance to individuals when their usual aides were in coach training.

Addressing these constraints, we developed the Stroke Homehealth Aide Recovery Program (SHARP) to prepare a select group of frontline HHAs to become “peer coaches” who would support other frontline aides in the field. Graduates of SHARP training were intended to serve as a mobile corps of coaches, coordinating with physical therapists and mentoring frontline aides. Mentorship was to include instruction and modeling of techniques that frontline aides could use to motivate patients, increase their physical activity, and promote greater adherence to prescribed home exercise regimens. The concept of a mobile coaching corps was appealing for several reasons. First, coaches would be readily available to guide and assist frontline aides on a “just in time” basis when recovery of skills impaired by stroke was most salient to an individual. Second, the skills acquired by the frontline aides were “multiplicative” in that they could be applied to future individuals served by a given aide. Third, the model would support workforce development by offering the prospect of career advancement not only to HHAs but also to aides in other rehabilitation settings where opportunities for advancement may be limited.

## Objectives

SHARP was conceived as a programmatic intervention intended to improve the mobility of individuals recovering from a stroke by enhancing the effectiveness of post-stroke home-based rehabilitation services. This was to be accomplished without adding costly new human resources by leveraging HHAs trained as stroke coaches to motivate increased frequency and intensification of individuals’ prescribed exercise regimens and overall level of activity. The goal of the pilot study was to assess the feasibility of the SHARP intervention and to pilot research procedures in anticipation of a full-scale randomized controlled effectiveness trial. The study’s specific objectives were to assess (1) the practicability of procedures for recruiting coaches and for enrolling and randomizing participants; (2) program acceptability to coaches, frontline HHAs, participants, and physical therapists; (3) fidelity to the SHARP intervention and study protocols; and (4) the performance of the study’s outcome measures.

## Methods

### Overview

Pilot studies are a subset of feasibility studies that ask the same question—can something be done and, if so, how—but also are designed to include a small-scale test of study procedures [33]. This study was a mixed methods feasibility study that included a pilot trial. The pilot study randomized 60 home health patients to either the intervention (SHARP + usual home care) or usual home care (UHC) only. We determined a priori that this sample size would suffice to allow examination of enrollment, deployment, and intervention processes, as well feasibility and variability of performance-based outcome measures over time [34]. Data sources included structured and semi-structured interviews and study records; data analysis consisted of both qualitative and quantitative techniques.

### Conceptual framework

The World Health Organization International Classification of Function (ICF) model [35] informed the study components and measure selection. The SHARP intervention was conceived as an *environmental factor*. Reinforcing physical exercise regimens targeted at muscle strength, coordination, and balance, it aimed to ameliorate impairments in *body function/structure* and in *activities* such as walking and transfers. In turn, the focus on improving individuals’ capacity to perform mobility-related activities was intended to allow for greater *social participation and improved quality of life.*

### Setting

The study was a collaboration between senior researchers and clinicians at a major academic health center and those at a large nonprofit Medicare-certified home health organization serving the five boroughs of New York City, with a population of 8.8 million people. All study participants had experienced an ischemic or hemorrhagic stroke within 90 days of study enrollment, and all received usual home care regardless of study arm. The study protocol was approved by the institutional review boards of the host organization and the academic partner involved in the study.

### SHARP intervention

#### Coaching

SHARP coaches were to occupy a dual role—they were trained as mentors to frontline aides serving newly enrolled study participants, while continuing as frontline aides to patients without stroke when not coaching. Joint *coach/HHA* visits were to be the venue for the coaching process. At these visits, coaches were to explain their role, model ways to encourage increased repetition of the participant’s prescribed exercise regimen and greater activity in general, observe the HHA with the participant, and provide constructive feedback. Support to the frontline HHA and indirectly to the patient/family caregiver was designed to enhance the patient’s post-stroke recovery through two main pathways: (1) patient-centered reinforcement of prescribed physical therapy exercises and (2) early recognition of exercise barriers—environmental, psychological, or family-related—that might be readily observed through daily HHA contact and that might addressed by the coach and frontline aide under the guidance of the physical therapist.

#### Visit protocol

The protocol specified a 30-day intervention period with four, 45–60 min in-home visits. To facilitate effective team coordination the coach’s first visit was to be conducted jointly with the physical therapist and the frontline aide in the participant’s home. At each of the subsequent three visits, the coach was to check in with the patient participant and the HHA regarding progress toward the mobility goal established at the prior visit, review the participant’s recent overall level of activity, discuss any barriers encountered, and work with the HHA and the participant to establish a new goal going forward. The coach also was to document the content of the visit and provide an oral report to study staff. Additional coaching visits and calls could be scheduled as needed depending on the frontline aide’s progress in addressing barriers and the participant’s progress toward mobility goals. The research team tracked all contacts for use in the fidelity assessment.

### Coach eligibility, identification, training, and selection

Trainee candidates were drawn from volunteers and recommendations of HHA field supervisors. Operations personnel of the collaborating home health aide division completed pre-screening of potential candidates. Pre-screening and final selection criteria appear in Table 1. It was expected that twenty HHAs would be needed to secure ten eligible candidates for training, of whom 80% would complete the training.

**Table 1:** SHARP coach trainee selection criteria

*Initial criteria to be selected for an interview (pre-screening)* ► At least 1 year of satisfactory prior employment at the home health agency ► Prior coach training (at the home health agency or elsewhere) comprising a minimum 1-week course including patient-centered techniques to support self-management through improved communication (e.g., reflective listening), joint goal setting, and motivational interviewing ► Recommended by the coach trainer or their direct supervisor as someone who has the following: ▪ Basic knowledge on assisting patients to appropriately transfer and ambulate ▪ A desire to learn ▪ A desire to promote patient independence and health ▪ Good interpersonal and communication skills	
*Criteria to be selected for coach training as assessed through an in-person interview (via structured questions, role play, hands-on demonstration)* ► Demonstrates a desire to learn ► Demonstrates sincere interest in helping others learn and grow ► Expresses comfort/empathy in working with patients who may have difficulty in communicating ► Strong listening and ability to project a nonjudgmental supportive presence ► Demonstrates problem-solving capacity ► Demonstrates good basic transferring and ambulation skills ► Knowledge of falls prevention strategies ► Demonstrates satisfactory level of writing and reading comprehension skills	

Training consisted of 5 days of didactic, role-playing, and “hands-on” sessions employing adult learning techniques, followed by a 1-day booster session. The topics included in phase 1, “basic,” and phase 2, “advanced,” are detailed in Table 2. Session instructors included senior faculty from the participating medical center, experts in cultural competency and motivational techniques, trainers for the rehabilitation department of the parent agency, and study staff.

**Table 2:** Stroke coach curriculum overview

Topic	Didactic (D); role playing (R); hands-on (H)
Phase 1: Basic sessions	
Stroke overview: causes and after-effects	D
Falls prevention: risk factors, environmental assessment, and balance/strength exercises to reduce risk	D, R
Cultural competency/sensitivity training	D, R
Review of common rehabilitation care plans	D, R
Depression/anxiety recognition	D, R
Role and participation in the rehabilitation team	D
Problem-solving	D, R
Phase 2: Advanced sessions	
Ambulation and transfer	D, R, H
Range of motion techniques for post-stroke patients	D, R, H
Speech/communication	D, R
Preventing another stroke through medication compliance and diet	D and video
Recognizing another stroke and appropriate response	D and video
Patient/family engagement, motivation, and goal setting (motivational interviewing coaching refresher)	D, R, H
Working synergistically with therapists and caregivers	D, R
Enhanced observe, record, report: recognizing and relaying signs of deteriorating conditions and other factors to prevent emergencies and unnecessary re-hospitalizations	D, R, H
Train-the-trainer techniques; teach-back techniques	D, R, H
Phase 3: Field observation and support	
Direct observation of peer education field visit	H
Booster sessions	D, R, H

To proceed as a SHARP coach, trainees had to pass a post-training written test and a role play observation qualifier. After deployment, each coach received field observation and immediate feedback plus a full debriefing after each completed case.

### Participant eligibility, identification, recruitment, and randomization

To participate in the study, patient participants were required to be:

1. 18 years of age or older
2. English-speaking
3. Post-stroke (within 90 days of start of home care services)
4. Receiving home health physical therapy services
5. Receiving HHA services
6. Without a diagnosis of Alzheimer’s or dementia
7. Able to provide informed consent as determined through a brief cognitive screen and fuller consent process
8. Able to walk independently or with non-contact assistance (see below)
9. Mobility impaired but requiring no or only minimal assistance and potential for improved mobility (see below)

The participants in the study were referred to home care with a diagnosis of “other sequelae of cerebral infarction.” Post-stroke rehabilitation potential and level of mobility were determined through four steps: (1) examination of the plan of care to ascertain that the prescribing physician was referring the individual to home care services to work on mobility, (2) examination of the medical record to rule out those who were permanently bed- or wheelchair-bound, (3) an email confirmation to the physical therapist that the individual was working on mobility improvement, and (4) a phone eligibility screen that asked potential participants about their current ability to walk and the type/amount of assistance they were receiving. These questions were developed using definitions taken from the Functional Independence Measure (FIM [36];). Individuals with levels of complete independence (no contact assistance) to minimal (contact) assistance were eligible. The use of gait aids was permitted.

The screening process was designed to identify 120 potentially eligible individuals, of whom 60 would be eligible and consent to be randomized to the intervention or control group. First, potentially eligible participants were identified through referrals from physical therapists and the application of an electronic screening algorithm to the host organization’s electronic health record database. Next, conducted via telephone, the research assistant determined if the individual had sufficient communication skills to participate in a telephone call. Then, the assistant administered a brief cognitive assessment to verify the individual’s ability to give informed consent [37], reviewed their mobility status, described the study, received verbal consent, and set up an in-home interview. At the in-home interview, a trained research interviewer explained the study again, received written consent, and proceeded to administer the baseline participant assessment instrument. After individual consent was received and the baseline assessment completed, participants were randomized. Block randomization with random block sizes of four and six was carried out to balance the intervention and usual care groups. A sequence of random assignments was generated by the study programmer and sealed in sequentially numbered envelopes. Participants were sequentially assigned to their group after completing the consent and initial home visit.

### Data sources

The study drew on 6 main data sources as follows:

1. Daily 4-question surveys provided information on trainees’ satisfaction with the content, structure, and delivery of each day’s training session. A longer pre/post-training survey was administered to assess trainees’ knowledge acquisition (data purpose: training acceptability and impact).
2. Electronic health records (EHRs) were the source of patient-level data used to identify potentially eligible individuals and provide information on their clinical and functional status. The EHR data were derived from the Outcomes Assessment and Information Set (OASIS), an assessment battery mandated by the federal government for all home care patients receiving Medicare services [38] (data purpose: eligibility screening, descriptive data on patient participants.)
3. Structured in-home interviews and mobility assessments were conducted at study enrollment and 60 days thereafter. The interviews were conducted by research interviewers blinded to the participant’s study arm and included, in addition to performance-based outcome measures, questions about their personal characteristics, prior health service use, health conditions, and functional dependencies. The instruments were pilot tested prior to study implementation to assess participant burden and identify areas that might be shortened to reduce that burden. All study participants received a $25 honorarium for each completed baseline and follow-up interview (data purpose: baseline and follow-up data to describe participant sample and assess the performance of study measures).
4. Semi-structured post-program telephone interviews were conducted with intervention and usual care participants. Administered by research staff, the interviews consisted of both objective and open-ended questions related to individuals’ perceptions of their stroke recovery and exercise regimen. Intervention participants also were asked specifically about their perceptions of the SHARP program and the assistance it provided (data purpose: intervention acceptability and participant perceptions of rehabilitation impact).
5. Semi-structured mid-program and post-program interviews with SHARP coaches, physical therapists, and frontline aides assessed the program acceptability from their respective points of view. The interviews, conducted by research staff, provided information on respondents’ experience, perceptions, and attitudes toward aspects of the SHARP intervention. Recommendations for future program modifications also were solicited (data purpose: intervention acceptability).
6. Enrollment and visit tracking reports, workflow information, regular check-in meetings between study staff and coaches to discuss caseload and progress, and audio recordings of coach visits allowed study staff to closely monitor program activities (data purpose: fidelity to intervention protocol and research procedures).

### Study measures

#### Individual-level outcome measures derived from structured interviews

The primary study outcome was mobility, measured by two performance-based mobility instruments: the 4-Meter Walk Gait Speed test [39, 40] (a Common Data Element recommended for research purposes by the National Institute of Neurological Disorders and Stroke (NINDS)) and the Timed Up and Go (TUG) [41–43]. Both tests have shown excellent validity and reliability [40–42] when administered to individuals post-stroke [44–46] and were reportedly easy to use. Covariate measures included a combination of validated OASIS and NINDS Common Data Elements measuring demographics (e.g., age, sex, race), clinical status (e.g., medical diagnoses such as heart failure, hypertension, arthritis), functional status (e.g., activities of daily living/instrumental activities of daily living [ADLs/IADLs]), and health-related quality of life.

#### Program acceptability measures

Training acceptability to *coaches* was measured daily. On a 4-point Likert scale ranging from “very much” to “not at all,” trainees responded to four statements about the clarity of information, the sufficient allotment of time to new information, the opportunity for discussion, and the helpfulness of handouts. Using a formal interview guide, semi-structured interviews with *coaches*, *physical therapists*, and *frontline aides* focused on (a) respondents’ experience/satisfaction with their preparation and role, (b) communications/coordination with other key members of the SHARP team, (c) perceived program strengths and weaknesses, and (d) recommendations for changes in areas such as training, communication, or visit protocols that could improve the program. The measures included in the semi-structured *post-program participant survey* included a question that asked individuals to rate on a scale from 0 to 100 their satisfaction with their stroke recovery. The survey also included open-ended questions that asked individuals about their physical therapy sessions, their daily exercise routines, the types of assistance provided by their aide, and the “things or services” that “may be helpful for your stroke recovery.” Intervention participants specifically were asked to rate their satisfaction with their coach on a 0 to 100 scale, to describe the types of assistance the SHARP coach provided and their experience in setting a rehabilitation goal, and to indicate why or why not they would recommend SHARP participation to another post-stroke patient.

#### Fidelity measures

We focused on three aspects of fidelity: *dose* or exposure to the intervention, *adherence* to key intervention components, and *content* of the intervention [47]. The primary measures of intervention fidelity were (1) percentage of the intervention group receiving all four planned coach visits (80% acceptability threshold) (dose and adherence), (2) percentage of expected joint coach/HHA sessions completed (80% acceptability threshold) (dose and adherence), (3) percentage of the intervention group with at least one collaborative coach/therapist visit (85% acceptability threshold) (dose and adherence), and (4) percentage of the intervention group with at least one mobility goal established (100% acceptability threshold) (content). Fidelity thresholds were based on the professional judgment of senior study staff and their assumptions about the importance of the measure to study outcomes and the feasibility of implementing the specific activity [48].

### Analysis

#### Structured data

Quantitative analysis of all structured data was mainly descriptive (summary measures of means, range, variance, and standard deviation). For individual-level survey measures, we also examined the correlations among covariate and outcome variables and unadjusted effect sizes for outcome measures with the caveat that the pilot—geared toward evaluating the response burden and variability to inform a larger study—was not powered to detect statistically significant outcomes within the study population.

#### Semi-structured data

Thematic analysis [49–51] was used to analyze the data from the semi-structured post-program interviews with coaches, HHAs, therapists, and participants; each group comprised a separate data set. The study staff used an atheoretical, inductive approach in reviewing data to identify themes and subthemes from interview participants’ explicit responses to open-ended questions. Statements about the perceived benefits/advantages/favorable aspects of SHARP or its disadvantages/difficulties/less favorable aspects were all included in the analysis of intervention acceptability. Once themes and subthemes were reviewed and refined, supporting quotes were extracted for illustrative purposes.

#### Study tracking reports, visit summaries, and workflow information

Analysis of these data was iterative and dynamic [52]. This approach was used to provide information allowing for midcourse changes in the research design or intervention protocol should they prove necessary.

## Results

### SHARP recruitment and randomization procedures

#### Coaches

HHA supervisors and training staff prescreened and referred 18 coach candidates to be interviewed for coach training. Of this group, 10 were selected for the 5-day SHARP training and nine were approved for deployment as coaches. However, due to unanticipated delays in participant recruitment and enrollment, seven of the nine coaches experienced job changes during the study period that either precluded or limited their availability to take SHARP cases. Thus, six coaches were deployed over the course of the intervention. Three were assigned to one participant each and three to four or more participants each. The main obstacles to full coach participation in the intervention stemmed from conflicts with a coach’s regularly scheduled HHA caseload, long travel time from one visit location to another, and the study’s target for completing the first coaching visit within 7 days of intervention assignment.

#### Participant enrollment and randomization

**Fig. 1:**
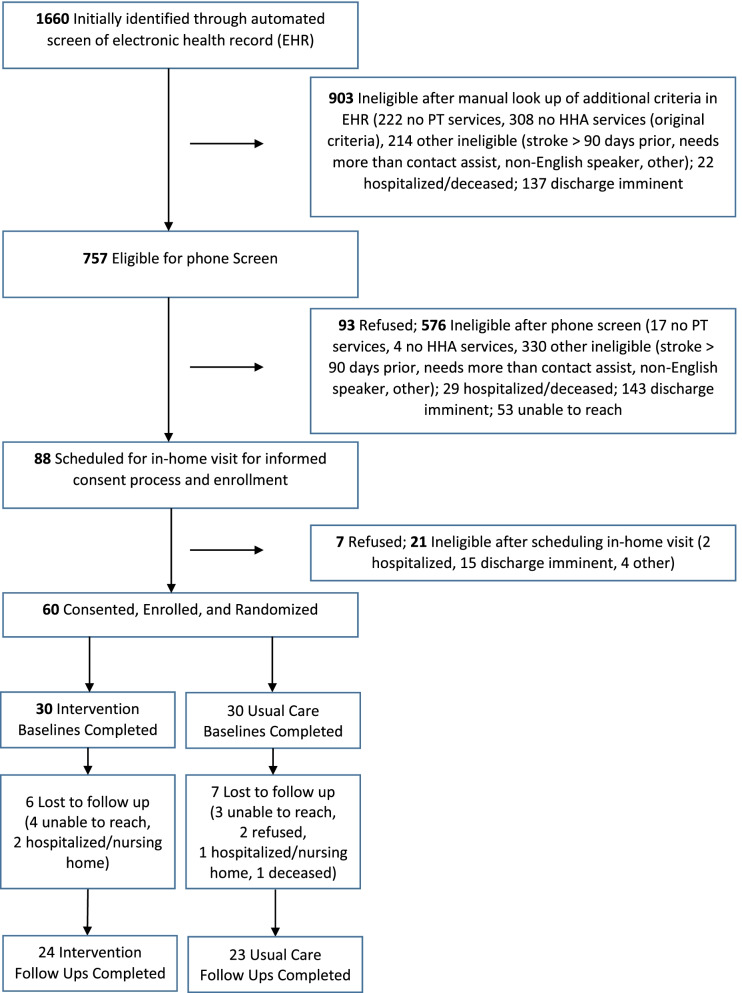
Consort diagram

Enrollment was conducted from June 2018 through May 2019 with all participant follow-up interviews completed by August 2019. Because the role of the SHARP coach was conceived as providing just-in-time peer mentorship to a participant’s frontline aide in the field, the eligibility criteria required that participants have HHA service as part of their plan of care. According to the projections of service delivery patterns prior to submitting the study’s grant proposal, 40% of patients were receiving HHA services; enrollment targets were based on those projections. However, during the subsequent enrollment period, only 20% were receiving HHA services—a result of changes in reimbursement, agency practice, and/or patient caseloads that were outside the control of study staff. Consequently, during the first 5 months of enrollment, 59.8% (312) of potentially eligible participants had to be dropped in stage 1 or 2 of the screening process because they had no HHA services (Fig. 1, Consort diagram). An additional enrollment challenge was the shortening of home health lengths of stay, which averaged 38 days when proposal estimates were prepared versus 34 days during the enrollment period. As a result, 18% of potentially eligible participants were excluded due to early or imminent discharge from physical therapy or the agency altogether.

To address the dearth of eligible participants and meet enrollment targets within the allotted time period, recruitment for the pilot was expanded from one New York City borough to four. Additionally, eligibility criteria were altered to remove the HHA service criterion. This, in turn, required a significant change to the intervention protocol, which was approved by the study’s NIH project officer as well as the respective IRBs. Under the revised protocol, individuals without an assigned HHA became study-eligible and were randomized to either the intervention or usual care group. Correspondingly, the role of SHARP coaches shifted from mentoring frontline HHAs to coaching study participants directly.

Of 1660 individuals who were screened, 88 were identified as eligible for an in-home visit to obtain formal consent and enrollment. The Consort diagram (Fig. 1) details the reasons for ineligibility. Of the 88 people designated for an in-home interview, 60 consented, enrolled, and were successfully randomized—30 each to the intervention and usual care groups. Forty-seven (78%) of the 60 completed the follow-up assessments, somewhat shy of the projected 85% retention rate (Fig. 1).

Table 3 summarizes the baseline characteristics of study participants with the data organized according to the Andersen health care utilization model [53]: predisposing characteristics (e.g., age, sex, race), enabling characteristics (e.g., education, income), and need/illness characteristics (e.g., chronic health conditions, stroke history, functional status). Table 3 also summarizes the participants’ scores on the study’s primary and secondary outcome measures. Participants’ average age was 65.6 years; the majority were female (60%); 51.6% were Black, and 30% Hispanic. A high school degree or less was the highest level of education achieved by 40% of participants; 28% had an annual family income of less than $25,000. All participants had experienced a stroke prior to enrollment, with an average of 47.6 days prior to baseline assessment; almost 40% had experienced two or more strokes. Eighty percent reported an ischemic stroke. The participants’ average score for the Timed Up and Go was 35 s and 11 s for the 4-Meter Walk Gait Test. As Table 3 indicates, the randomized groups were well balanced at baseline.

**Table 3:** Patient characteristics

	Total (*N* = 60)	Usual care (*N* = 30)	Intervention (*N* = 30)
Predisposing patient characteristics			
Age (mean, SD)	65.6 (13.2)	65.2 (13.5)	66.0 (13.0)
Female (%)	60.0	63.3	56.7
Race (%)			
Black/African-American	51.6	53.3	50.0
White	21.7	16.7	26.7
Others/not specified	26.6	30.0	23.3
Hispanic (%)	30.0	36.7	23.3
*Marital status (%)*			
Single/never married	26.7	23.3	30.0
Married/domestic partnership	43.3	43.3	43.3
Divorced/separated	8.3	6.7	10.0
Widowed	21.7	26.7	16.6
Enabling patient characteristics			
*Education (%)*			
8th grade or less	6.7	6.7	6.7
Some high school, but did not graduate	16.7	20.0	13.3
High school or GED	16.7	20.0	13.3
Some college or 2-year degree	36.7	36.7	36.7
4-year college graduate	8.3	6.7	10.0
More than 4-year college degree	15.0	10.0	20.0
*Income (%)*			
$0 to $9999 annually	10.0	3.3	16.7
$10,000 to $14,999 annually	8.3	6.7	10.0
$15,000 to $24,999 annually	10.0	16.7	3.3
$25,000 to $34,999 annually	16.7	23.3	10.0
$35,000 to $49,999 annually	11.7	6.7	16.7
$50,000 to 75,000 annually	20.0	16.7	23.3
$75,000 and above annually	11.7	13.3	10.0
Unknown/refusal	11.7	13.3	10.0
Baseline need/illness level characteristics			
*Health conditions (%)*			
No. of co-morbidities (mean, SD)	2.5 (1.4)	2.6 (1.6)	2.5 (1.2)
*Barthel Functional Status* (mean, SD) (higher scores = lower functioning)	78.0 (17.8)	77.3 (18.4)	78.7 (15.5)
*Falls risk conditions (%)*			
Two or more falls in the previous 12 months	40.0	46.7	33.3
Incontinence	40.0	43.3	36.7
Dizziness on standing up	45.0	53.3	36.7
*Stroke history*			
Two or more strokes (%)	36.7	43.3	29.6
Most recent stroke, type report in the medical record (%)			
Ischemic	80.0	73.3	86.7
Hemorrhagic	11.7	16.7	6.7
Unable to classify	8.3	10.0	6.7
Time since most recent stroke/TIA (days, mean, SD)	47.6 (25.4)	45.1 (27.8)	50.0 (22.9)
Baseline performance measures			
*Primary mobility measures*			
Timed Up and Go, s (mean, SD)	34.99 (21.9)	37.31 (22.2)	32.76 (21.7)
4-Meter Walk Gait Test, s (mean, SD)	11.10 (5.9)	10.86 (5.2)	11.34 (6.6)
*Secondary performance measures*			
30-Second Chair Stand, s (mean, SD)	3.98 (3.5)	3.54 (3.9)	4.38 (3.2)
4-Stage Balance Test, s (mean, SD)	22.79 (10.8)	21.81 (11.1)	23.67 (10.6)

*SD* standard deviation

### SHARP acceptability

#### Coaches

All ten coach trainees were asked to complete surveys on their satisfaction with the training program immediately on completion of the training. Mid-program and post-program interviews were conducted with only the three coaches who worked with four or more participants each. According to daily evaluations, the ten coach trainees were highly satisfied with their training. Trainees indicated that the training “very much” met their needs 98% of the time as assessed through four training-related statements over the 5-day training period. Analysis of mid-program and post-program interviews with the three coaches who worked with four or more participants identified five main themes.

1. Training and preparedness: Although coaches were very satisfied with their training, they felt they needed additional one on one contact with study staff. Especially useful were the required “report backs” to study staff after each coaching visit, which provided helpful ideas for motivating participants and helping them formulate exercise goals.
  - Coach 1: “the training from the PTs and doctors, it was great…very informative, very detailed. I would have liked more practice…just one-to-one to review things like patients get stuck on their goals.”
  - Coach 2: “…calling in [to give an update about the visit], which I think was great. It kept me in touch …. kept the case on track. Verbally going through it again – thought it was great.”
  - Coach 3: “the one on one training was good, I liked that. I liked reaching out to report the patient visits was great. I thought that was helpful.”
2. Interactions with patients/families: Coaches felt that patients and families were surprisingly open with them. Introduced as coaches, they felt they received greater respect and recognition from patients than when they worked as a frontline aide.
  - Coach 1: “I wasn’t expecting how open some patients would be right away, and that was inspiring. Patients opened up about…what they were going through with their families.”
  - Coach 2: “It was great to interact with patients and see how they’re doing. Encouraging them felt good.”
  - Coach 3: “I didn’t expect to interact so much……It was new for patients – being asked rather than told what to work on. Some patients really like being asked.”
3. Challenges of goal setting: Despite newly acquired skills, the coaches sometimes struggled with finding effective ways to help patients set realistic goals that were both motivating and achievable.
  - Coach 1: “…when you come and they weren’t able to meet the goal, it can be upsetting, and challenging.”
  - Coach 2: “…sometimes it feels like you’re talking to them, but you can’t get through to them. It’s like you try, you try to talk to them, but they’re stuck, they don’t want to do anything.”
  - Coach 3: “Patients that had pain…it restricted them from meeting or working on their goal.”
4. Rewards of goal setting: The coaches understood that their role was to motivate patients to practice their exercises and be as active as they could. When the patient established a mobility goal and was able to reach it, that was gratifying and motivating for the coach as well as the patient.
  - Coach 1: “When you come and they’ve been able to meet the goal, it’s a good feeling….”
  - Coach 2: “Once they realized the difference and they felt the difference. I think they realized that the mobility goals – like being more active – was really helpful.”
  - Coach 3: “…first patient was an elderly gentleman who had to walk previously with a walker. So he had the stroke and he had difficulty walking…but he was very much into exercise…and he was keeping up with that. So he was excellent….he knew that integrating mobility goals was very important so he kept that up.”
5. Patient supports: The coaches perceived that strong family support and encouragement facilitated their role.
  - Coach 1: “When they’re alone, [the patients] usually feel sad and very depressed…. People with a good support system… they’re doing much more and they recover faster.”
  - Coach 2: “Patients with good caregivers and more support …more likely to be successful.”
  - Coach 3: “People who doesn’t have any support…. [that’s] a problem.”

#### Physical therapists

Rehabilitation supervisors and training staff enthusiastically participated in planning the SHARP intervention, and the training staff were directly involved in the coach training sessions. To engage field therapists in the program, senior research staff attended two therapy team meetings, described the SHARP intervention, elicited therapists’ thoughts, and responded to their questions. Research staff also made calls to individual therapists to tell them that one of their patients was participating in SHARP and to provide additional information about the program. Thirteen of the twenty-seven field therapists whose patient had a SHARP coach responded to interview requests and participated in post-program interviews. Eleven of the 13 made a positive comment about SHARP, but only two did so in the context of a specific patient. The others, who often were uncertain about whether or not their patient had a SHARP coach, made more abstract comments about the value of the program.

The following are about a specific patient:

- PT 1: “…the patient used to nap a lot and complain that he was often tired, after the introduction of the SHARP coach, this improved…. staff were very reachable. If I needed to speak to someone, I could do it right away.”
- PT 2: “…I noticed that my patient was more motivated and active after beginning the SHARP project.”

The following are general comments:

- PT 3: “…the program has a great purpose…”
- PT 11: “HHAs are a valuable and untapped resource.”
- PT 13: “I like the idea of the project. It’s a good idea to reinforce the PT and establish consistency with the patient, sending a consistent message.”
- PT 7: “…it’s a wonderful idea…another bridge or connection to patient education and reinforcement.”

Respondents also made suggestions about how the program could be improved by changing the timing of joint visits and giving the coach more flexibility to accommodate the therapist’s schedule:

- PT15: “…the joint visit would be most beneficial to be toward the middle/end of the patient’s PT time, this would give the PT a chance to really work with the patient and get to know them and then also give the coach a chance to get to know the patient and address any issues with the PT before [the patient was] discharged.”
- PT 9: “I think the joint visit is a good idea but would not be feasible unless the coach was very flexible….sometimes this could mean waiting an hour or two at the patient’s house for the PT to arrive if the prior visit runs longer or there is traffic or parking issues.”

Most of the therapists who were interviewed had only a vague recollection of emails or calls from project staff and were unsure if one of their patients had a SHARP coach.

- PT 5: “…I remember getting a call that my patient was in the program, but I’m not sure when the coaching started, and I haven’t seen any changes in my patient that I would tie to the coach.”
- PT 12: “I remember getting a call that my patient was in the program...not sure when the coaching started… [I] haven’t seen any changes in my patient.”
- PT 8: “…neither of my patients talked about it ….[I] didn’t see any specific changes I could identify.”

The therapists cited heavy caseloads and busy schedules as obstacles to their active participation:

- PT 4: “Sometimes I have a caseload of more than 15 patients a day….It’s frustrating.”
- PT 10: “The PTs are far too busy and cannot be reaching out to you [i.e., SHARP staff] about the project.”

#### Frontline HHAs

Ten of the 30 individuals in the SHARP intervention group had a frontline HHA present for at least one coach visit; the study staff were able to complete a post-program interview with three HHAs. Those who were interviewed did not perceive that the objectives of SHARP fit with their defined responsibilities. One of the three was positive about the program, but the other two were not. The latter said they would be happy to help if the patient requested an exercise reminder or other exercise assistance. However, they did not view it as their role to initiate or encourage such activity without specific authorization in the formal plan of care signed by the patient’s physician.

- Frontline aide 1: “…[coach] was nice, informative, and patient.”
- Frontline aide 2: “[helping patient with exercises] …not on my care plan.”
- Frontline aide 3: “…can’t do things that [are] not on [my] care plan.”

#### Patient participants

Study staff completed 36 post-program interviews divided evenly between intervention and UHC participants. On a scale from 0 to 100, intervention participants reported greater satisfaction with their stroke recovery than UHC participants (71.9 [16.8] vs 53.8 [21.7]). When questioned directly about their experience with SHARP, intervention participants rated their satisfaction with their SHARP coach at an average of 95 on a scale of 0–100.

Open-ended questions asked participants in both groups if they remembered what exercises they were asked to do and what they did between physical therapy appointments. Participants also were asked if anyone helped them with their exercises and if they set any goals to increase their level of activity. We identified three main themes in participants’ responses to these questions:

1. Confidence and encouragement: The word confidence occurred in 11 of the 36 participant interviews and was almost always associated with belief by participants that they had the capability to do more than they thought they could. The importance of confidence was more often mentioned by intervention participants than by those in the usual care group. Among intervention participants, the coach was usually the source of encouragement, while among those in usual care it was usually the HHA or the therapist:
  - Participant 1 (SHARP): “[My] coach was very good…. [Coach’s name] gave me confidence and answered my questions. [I] worked with her every week…. It was a boost.”
  - Participant 7 (SHARP): “[Coach’s name] was kind and understanding, especially when I wasn’t feeling motivated. Now I can walk several blocks around my neighborhood and it’s all because of [Coach’s name].”
  - Participant 4 (usual care): “[HHA name] is very encouraging. Until today I couldn’t get in and out of the tub alone, but today I could do it.”
2. Goal setting and accountability: More than half of the participants interviewed commented explicitly on the goals they set while receiving physical therapy. Among intervention participants, sixteen talked about goals, and among usual care participants, four. Physical therapists played a prominent role in both groups, but in the intervention group, the SHARP coach was most often mentioned as the one who asked participants what they wanted to accomplish in the coming week and helped in setting a realistic goal. In the usual care group, it was usually the physical therapist. Goals ranged from repeating an exercise more frequently to climbing a stairway to venturing onto the subway. And once a goal was agreed on, participants did not want to disappoint the coach, the therapist, or themselves by not accomplishing it.
  - Participant 2 (SHARP): “Without [coach’s name] I probably wouldn’t be exercising today….she was a little naggy but it worked….My goal was increasing my exercise ‘reps’ from 15 to 18, and now it’s 20. Before [coach’s name] came, no one asked me if I didn’t do them.”
  - Participant 20 (SHARP): “If I started out strong for the week with my goal and didn’t keep up the momentum, I’d be a little down and I’d be discouraged and not work so hard. But [Coach’s name] gave me a little bit of a push and it helped.”
  - Participant 30 (SHARP): “[Coach’s name] would sit down with me and we would talk about my goal for the week. I could set my own goal, and we’d sit down together and schedule the week and fill out the worksheet.”
  - Participant 15 (usual care): “My PT set the goals. They’re the experts and they should be telling me what to do. I wouldn’t have felt comfortable telling the PT what my goal is and I don’t think my opinions should count because I don’t know. I’m not an expert.”
3. General support and positive reinforcement: SHARP participants commented that they appreciated their coach’s overall support and enthusiasm when they met a goal—whether it was as modest as increasing the number of times they repeated an exercise or as ambitious as walking around in the neighborhood.
  - Participant 11 (SHARP): “[Coach’s name] was all around positive and helpful. I rate her 100%, you can’t get any higher than that. She is pleasant and supportive and gave me information about how to identify the signs of a stroke.”
  - Participant 17 (SHARP): “I love her – [Coach’s name]! She’s friendly and she asks questions. With her suggestions I increase my level of activity each week. I’m walking in the hallway, and I’m walking outside. I wish I could go to my gym. My wife took two months off from work to help me. She’s my biggest supporter with my exercises. She reminds me and encourages me to do them. Now I’m able to get around more.”
  - Participant 11 (usual care): “I’m working on walking around my house without my walker, but I don’t feel safe walking outside. I ask my son for help if I feel an exercise isn’t safe. My son is a good support.”

### SHARP intervention fidelity

#### Overview

Intervention fidelity results were mixed. Coaches exceeded the pre-established 80% threshold for planned visits to participants’ homes. Additionally, coaches and participants met the 100% threshold for setting at least one mobility exercise goal over the course of the intervention. In contrast, the study failed to meet either the 80% threshold for joint coach/HHA visits or the 85% threshold for joint coach/physical therapist visits. Table 4 summarizes fidelity findings according to the four metrics established at the start of the study.

**Table 4:** Intervention fidelity metrics

Primary metrics	Percent	Notes
% of cases, in which the four protocol visits were completed	87%	This metric counts in-person visits; two out of thirty patients never started the intervention, two did not have four in-person visits.
% of cases with a joint health coach/field HHA visit	33%	This includes cases in which the coach directly worked with the aide and cases in which the aide observed the coach interaction with the patient.
% of cases with a joint health coach/physical therapist visit	0	Among six coaches and twenty-seven physical therapists, there were no joint visits.
% of cases in which at least 1 SMART mobility goal was established	100%	28 is the number of eligible cases, two did not move forward with visit 1.

#### Visit protocol (fidelity threshold 80%)

Twenty-six of the 30 intervention participants (87%) received all four required coach visits; no one received more than four, even though coaches had the discretion to schedule more. Of the four participants who did not receive all four visits, one could not be reached, one was hospitalized and then discharged to a nursing home, and two moved (one due to a fire) to a new residence where they could not receive an outside visitor. Of a total of 110 coach visits that occurred, 45 (41%) included just the coach and patient, 32 (29%) included a family member, 24 (22%) included an aide, and 9 (8%) included both an aide and a family member.

#### Coach/HHA visits (fidelity threshold 80%)

The joint coach/HHA visit threshold was altered when the study protocol was changed to include patients without HHA services and the role of the coach correspondingly shifted from directly supporting the HHA to directly supporting the patient. Although HHAs were no longer the primary focus, coaches were still encouraged to engage them when their schedules coincided. Similarly, the coaches were encouraged to engage family members when they were present and willing. Ten SHARP participants (33%) had an HHA present for at least one coach visit (a total of 33 visits). A family member was present along with the HHA on four of these visits, and family members were involved on 12 other visits as well (a total of 41 visits). At eight visits (27%), there was never an aide or family member present (a total of 45 visits). Fixed and inflexible work schedules were the main obstacle to face-to-face coach/HHA contact. This was a problem for frontline HHAs, who nearly always were serving more than one patient, but also for the coaches, who in their dual role served as frontline HHAs in project downtime. Once the latter had an established HHA caseload, their schedules, too, were often inflexible.

#### Coach/physical therapist visits (85% fidelity threshold)

After attempting to schedule a joint coach/PT visit with the first three participants randomized to the intervention arm and further discussion with operations staff, the joint visit effort was halted due to substantial scheduling challenges. As noted above, coaches’ dual role as both coach and HHA meant that it was difficult for them to schedule a visit at a time convenient to both the patient and therapist. While participants were generally at home and available, physical therapists had tight schedules and were unresponsive to requests for schedule accommodation.

#### Goal setting (fidelity threshold one goal per intervention participant)

Coaches were able to obtain an audio recording for 65% of their coaching visits. The review of the recordings indicated that with the help of coaches all intervention participants were able to establish at least one mobility goal, albeit sometimes unrealistically ambitious. The audio recordings also showed that coaches who worked with at least two participants were more successful in establishing serial goals, monitoring an individual’s progress, and establishing new goals as appropriate. The ongoing research staff support was necessary to help coaches achieve a balance between maintaining participant rapport on the one hand and focusing on mobility goals on the other.

### Feasibility of selected study measures

The study’s primary outcome was change in mobility, measured by two performance measures: the Timed Up and Go (TUG) and the 4-Meter Walk Gait Speed Test. At the phone screen, all participants were alerted of the performance assessments and agreed to attempt them. At baseline, 16.6% did not complete one or both mobility measures. At follow-up, 25.5% did not complete one or both. Reasons for non-completion at baseline or follow-up included lack of unimpeded 4-m space or enough space to guard safely (13.1%), recent fall or general hesitance (8.4%), inappropriate chair (3.7%), and unavailable for in-person interview (1.9%). Accounting for study attrition, only 55% of participants completed both mobility measures at both baseline and follow-up. The main secondary outcome was fall risk. Similar rates of completion were seen with these performance measures.

### Participant outcome estimates

Because the data were incomplete and the pilot study was not powered to detect statistically significant differences between the randomized groups, we examined only the unadjusted results for the study’s two primary outcome measures. We found that the full study group reduced its mean baseline TUG score of 34.99 seconds (SD 21.9; range 11–104) by .5 s. The usual care group increased its mean baseline score by 6.5 s, while the intervention group decreased its mean baseline score by 4.5 s. The 4-Meter Walk Gait Speed measure yielded opposite results. The full study group reduced its mean baseline score of 11.10 seconds (SD 5.9; range 4–30) by .44 s. However, the usual care group got .31 s slower between baseline and follow-up, while the SHARP group got 1.14 s faster. In sum, the two measures yielded conflicting results. Because the pilot was not powered to detect statistically significant outcomes within the study population and because complete outcome measures were available for only 55% of participants, these findings should not be construed as clinically meaningful or reliable.

## Discussion

Research on both physical activity interventions [54–56] and intervention feasibility emphasizes the value of pilot testing complex interventions under conditions that closely mimic those of the eventual definitive study [33, 57]. The pilot test of SHARP was conducted under organizational circumstances that “mimicked” those of an eventual trial. Under these conditions, some parts of the intervention were successfully implemented. However, the study identified three substantial barriers to successful real-world implementation. Therefore, the significant redesign would be required before progressing to a definitive trial.

The pilot study successfully (1) recruited, trained, and graduated a slightly higher number of SHARP coaches than targeted (9 of 10 vs 8 of 10); (2) enrolled the targeted number of 60 participants with a well-balanced randomization between intervention and usual care groups; (3) completed planned baseline and 60-day follow-up in-person interviews, with a 60-day participant retention rate of 78%; (4) achieved high coach and participant satisfaction; and (5) produced rich information on which parts of the intervention “worked” and which did not. The three major implementation barriers it identified were (1) an eligibility criterion that impeded participant recruitment because it required an individual receiving post-stroke services to have an assigned frontline aide in order to become study-eligible, (2) a fidelity threshold that was not achieved because it required joint coach/therapist face-to-face meetings that were not scheduled, and (3) a primary outcome assessment that could not be completed for many participants because it relied on two performance-based mobility measures that were difficult to administer in the homes of study participants. The study’s main implementation obstacles, discussed below, stemmed from secular changes in service delivery, organizational constraints and workforce norms, and factors associated with individual circumstances. In this respect, the SHARP intervention encountered complexities observed in other pragmatic trials [58–69], including studies designed to move exercise interventions from tightly controlled organizational settings into the home and community. These complexities involve interactions among physical, behavioral, and environmental factors, and among service organizations, rehabilitation professionals, paraprofessionals, program participants, and their families [54, 55].

The main impediment to SHARP recruitment efforts was a steep decline in the assignment of HHAs to individuals receiving home health services for post-acute recovery. Between the planning period and study startup, the proportion of individuals receiving post-stroke HHA services at the host organization declined from 40 to 20%. As a result, nearly 60% of potentially eligible participants were ineligible in the first 5 months of study recruitment. This change, precipitated by changes in the external service delivery system, was not anticipated by rehabilitation managers or researchers. The research team had based the study’s enrollment projections on 5 years of prior data that showed a steady rate of HHA service use. Had they projected a less stable trend in the future, they could have considered alternative participant eligibility criteria or a change in pilot study design. Post hoc, the research team modified the study eligibility criteria and redesigned the intervention so that coaches could work directly with participants rather than with their HHAs. However, this modification represented a fundamental change in the conception of coaches as HHA peer mentors and altered the career advancement opportunity envisioned. It also reduced the multiplicative impact of the intervention because mentored HHAs potentially could have extended their newly acquired mobility reinforcement skills to subsequent individuals receiving their services.

The overly optimistic enrollment projections and slower than projected study enrollment that hampered SHARP recruitment are well-documented phenomena that have plagued many pragmatic effectiveness trials [59–69]. Recruitment problems in physical activity studies have been attributed to delays in screening, causing some potential participants to “time out” (e.g., exceed a designated number of weeks before study entry); the overly restrictive safety criteria that excluded potential participants who could have participated in an intervention without harm; low consent rates and difficulty finding control group participants who had not already been exposed to a comparable intervention [54, 55, 70]. Much has been written about improving recruitment by sending flyers and making telephone calls to clinicians and potential participants, assigning dedicated recruiters, offering participation incentives, and building trust in target communities [65, 71]. However, the principal barrier to SHARP recruitment—an unexpected change in the pattern of HHA service utilization—was a systems issue that the recruitment literature has rarely addressed [65, 69]. Little has been written about ways to adapt a pilot study to a dynamic service environment [65, 72, 73], the pros and cons of a three-arm pilot [74], or the implications of substituting an alternative intervention model for the control arm in a pilot study. These topics could be potentially fruitful areas for future research and design of pilot study protocols.

While study staff instituted changes in eligibility criteria and program design to enable the SHARP intervention to proceed in a different configuration, they did not rescue the care coordination component of the intervention. The joint in-home therapist/coach visit, intended to be the key mechanism for achieving coordination among coaches, physical therapists, HHAs, and patients, was a failure. Most of the obstacles to scheduling those visits could be attributed to organizational constraints and workforce norms. The host organization’s budget limitations required that coaches serve a dual role as coach and direct care HHA (whose visits, unlike the coaches’ visits, contribute to service revenues). However, direct care HHAs are subject to relatively inflexible service delivery schedules designed to accommodate patient needs. Therefore, coaches had little flexibility to coordinate their coaching visits with the visits of the physical therapist. Therapists’ heavy caseloads and other competing organizational requirements (e.g., training and orientation to a new electronic health record) also negatively affected intervention delivery. The workforce norm [75] by which rehabilitation therapists operated as autonomous professionals independent of HHAs in the field most likely compounded the problem. Related to this may have been professional bias underestimating the potential contributions of a paraprofessional coach to exercises and mobility goals established for an intervention group participant.

To increase therapists’ engagement with SHARP, the research team tried to strengthen communication channels. They attended therapists’ staff meetings to explain and promote the program and sent follow-up program materials. They also called individual therapists to confirm the participation of one of their patients and to give them additional information about the program. These communications, however, were only vaguely recalled by therapists and did not facilitate the successful scheduling of joint visits. Eliminating the coaches’ dual role and/or reducing the size of therapist caseloads, which possibly would have facilitated coordination but also would have been costly to the host organization, were outside the authority of the research team. The use of sophisticated but costly technology to convene virtual meetings between therapists and coaches was not considered by the study investigators. With the experience of COVID-19 and the acquisition by the host organization of sophisticated virtual technology for widespread use in the organization, the option for virtual coordination would likely become more feasible for a future study, as the costs could be attributed to general organizational infrastructure and overhead.

The SHARP experience reinforces the body of evidence on the implementation of complex interventions and pragmatic intervention trials. Evidence shows that these are especially vulnerable to multiple and varied organizational and structural obstacles that can delay, undermine or halt an otherwise promising study [59, 60, 76]. Studies of care coordination and collaboration also are replete with examples of organizational obstacles that impede cooperation [77, 78]. Ambiguity of professional roles, time constraints, resource limitations, logistical, and scheduling issues are among the impediments most frequently cited [78]. Among proposed remedies are enhanced resources, improved infrastructure, clearer role definitions, better communication channels, and material incentives for coordination [78]. SHARP researchers applied a few of these (e.g., role clarification and increased communication), with little effect. Among the structural factors shown to facilitate the adoption and implementation of complex interventions are clearly observable advantages in efficiency, effectiveness, or cost-effectiveness relative to current conditions; compatibility with existing organizational norms and operational procedures; the possibility for “reinvention” or modification by potential adopters; and the potential to improve the workability of essential tasks performed by those adopters [79–81]. As an intervention pilot, however, SHARP did not have data to demonstrate relative advantage. Further, to improve the potential of HHAs and improve the outcomes of individuals recovering from stroke, it was designed in part to disrupt rather than reinforce existing organizational norms and procedures. Thus, SHARP embodied an essential paradox of both pilot studies and larger pragmatic trials—that the evidence of effectiveness required to justify organizational investments or disruptive changes is often the evidence that must be collected by the study, in the absence of optimal intervention implementation.

Yet, another SHARP implementation barrier stemmed from federal regulations and associated norms and procedures at the host organization. These, in turn, affected the attitudes of frontline aides toward their role. Medicare payment and regulatory procedures are closely tied to a patient’s plan of care, which originates with the person’s referring physician and dictates the tasks to be performed by the HHA. These are typically routine tasks related to personal care and household chores that a patient cannot do due to limited capacity to carry out activities of daily living. Absent a specific request from the patient or family caregiver, frontline HHAs—who did not see exercise appear on the patient’s plan of care—felt it was “stepping out” of their role to suggest that an individual set a mobility goal or simply become more active. Any larger or longer-term SHARP effort thus would require high-level organizational support to enlist physicians and physical therapists to incorporate exercise support and repetition into the HHA’s responsibilities as outlined in the formal patient care plan.

Lastly, constraints imposed by participants’ personal circumstances and living conditions impeded the ability of researchers to measure SHARP outcomes. At the recommendation of stroke rehabilitation experts, the pilot study tested two validated outcome measures to assess its primary mobility outcome (the Timed UP and Go and the 4-Meter Walk Gait Speed Test). Both measures were among the inventory of performance tools used by physical therapists at the host organization, although no quantitative information was available on the frequency of their use. One of the purposes of the pilot was to assess their feasibility in the study population targeted for the SHARP intervention. Although the two measures were described to participants in their screening interview, in the field, we found that administering them often was problematic due to study participants’ living conditions, which often included small spaces, limited furniture choices and residence in the home or apartment of a family member or friend. Adding in the effect of participant attrition due to hospitalization/nursing home admission and inability to make contact, only 55% of participants completed both mobility measures at baseline and follow-up. For a future study to be successful, investigators would likely have to find one or more valid, reliable non-performance-based measures that could serve as an acceptable alternative to the measures tested in this study.

## Limitations

This study had several limitations. First, it was conducted in a single large organization with multiple layers of management responsible for overseeing care delivery to participants widely dispersed across New York City. Most home care organizations are much smaller and serve many fewer participants, with fewer management layers to oversee services in a much smaller catchment area. It is possible that a smaller organization could have provided an environment more conducive to intervention fidelity. Nevertheless, to achieve scale, any large RCT would have to engage organizations such as SHARP’s host and could well have encountered unforeseen obstacles such as those that led to intervention modification in this pilot. Second, it could be argued that the pilot test of a SHARP program with modified eligibility criteria resulting in significant program redesign was not in fact a fair test of the SHARP intervention. However, we would argue that we learned more about what “worked” and what did not by redesigning the intervention and moving forward than by abandoning the intervention and the pilot altogether. Third, few HHAs were available for program acceptability interviews—mostly because so few HHAs experienced what the SHARP coaches had to offer. Thus, we may have overstated the aides’ lack of enthusiasm for collaborating with coaches to reinforce participants’ adherence to repetitive exercise regimens. That said, the plan of care issues identified through the HHA interviews introduced an unforeseen regulatory factor that would have to be accounted for in any future test of a similar intervention. Lastly, the disconnect we observed between the positive comments of the thirteen therapists who participated in program acceptability interviews and their lack of clarity about SHARP specifics or coach/patient ties requires further investigation. Exploration of more robust communication methods and more workable coordination mechanisms is warranted, including the use of technology to increase program effectiveness.

## Conclusions

The SHARP intervention is not feasible in its current design. It failed to achieve its main objectives of training and deploying stroke coaches as peer mentors to frontline HHAs; coordinating the activities of coaches, HHAs, and physical therapists; gaining the support of key program participants beyond coaches and individuals in the intervention group; and accurately measuring primary outcomes. Combined, these impediments to implementing key components of the intervention mitigate against the expansion of SHARP in its current form. The study underscores the value of using pilot studies to assess participant recruitment, intervention acceptability, and fidelity to intervention protocols, as well the practicability of research procedures, including methods for participant randomization, data collection, and implementation of primary outcome measures. The study also highlights the potential benefits and limits of using implementation science instruments to anticipate environmental and organizational barriers to implementation and dissemination [82, 83]. In conclusion, better contingency planning and significant design changes—especially reconfiguring the role of coaches vis a vis frontline aides and patients, resolving communication and coordination problems affecting therapists and coaches, and identifying rigorous but practical outcome measures—would be necessary to make a modified SHARP program feasible for a definitive effectiveness study.

## Data Availability

The datasets used and analyzed during this study are available from the corresponding author on reasonable request.
